# Simplagrin, a Platelet Aggregation Inhibitor from *Simulium nigrimanum* Salivary Glands Specifically Binds to the Von Willebrand Factor Receptor in Collagen and Inhibits Carotid Thrombus Formation *In Vivo*

**DOI:** 10.1371/journal.pntd.0002947

**Published:** 2014-06-12

**Authors:** Andrezza C. Chagas, Peter McPhie, Hong San, David Narum, Karine Reiter, Fuyuki Tokomasu, Fabio A. Brayner, Luiz C. Alves, José M. C. Ribeiro, Eric Calvo

**Affiliations:** 1 Laboratory of Malaria and Vector Research, National Institute of Allergy and Infectious Diseases (NIAID), National Institutes of Health (NIH), Rockville, Maryland, United States of America; 2 Physical and Biochemistry Section, National Institute of Diabetes and Digestive and Kidney Diseases, NIH, Bethesda, Maryland, United States of America; 3 Animal Surgery and Resources Core, National Heart Lung and Blood Institute, NIH, Bethesda, Maryland, United States of America; 4 Laboratory of Malaria Immunology and Vaccinology, NIAID, NIH, Bethesda, Maryland, United States of America; 5 Centro de Pesquisas Aggeu Magalhães (CPqAM/FIOCRUZ) and Laboratório de Imunopatologia Keizo Asami. Universidade Federal de Pernambuco, Recife, Pernambuco, Brazil; Johns Hopkins Bloomberg School of Public Health, United States of America

## Abstract

**Background:**

Among the several challenges faced by bloodsucking arthropods, the vertebrate hemostatic response against blood loss represents an important barrier to efficient blood feeding. Here we report the first inhibitor of collagen-induced platelet aggregation derived from the salivary glands of a black fly (*Simulium nigrimanum*), named Simplagrin.

**Methods and Findings:**

Simplagrin was expressed in mammalian cells and purified by affinity-and size-exclusion chromatography. Light-scattering studies showed that Simplagrin has an elongated monomeric form with a hydrodynamic radius of 5.6 nm. Simplagrin binds to collagen (type I-VI) with high affinity (2–15 nM), and this interaction does not involve any significant conformational change as determined by circular dichroism spectroscopy. Simplagrin-collagen interaction is both entropically and enthalpically driven with a large negative ΔG, indicating that this interaction is favorable and occurs spontaneously. Simplagrin specifically inhibits von Willebrand factor interaction with collagen type III and completely blocks platelet adhesion to collagen under flow conditions at high shear rates; however, Simplagrin failed to block glycoprotein VI and Iα_2_β_1_ interaction to collagen. Simplagrin binds to RGQOGVMGF peptide with an affinity (K_D_ 11 nM) similar to that of Simplagrin for collagen. Furthermore, Simplagrin prevents laser-induced carotid thrombus formation *in vivo* without significant bleeding in mice and could be useful as an antithrombotic agent in thrombosis related disease.

**Conclusion:**

Our results support the orthology of the Aegyptin clade in bloodsucking Nematocera and the hypothesis of a faster evolutionary rate of salivary function of proteins from blood feeding arthropods.

## Author Summary

Blood feeding arthropods—like mosquitoes and black flies—have evolved salivary secretions rich in molecules that affect hemostasis, including vasodilators and inhibitors of blood clotting and platelet aggregation. Among the platelet inhibitors, antagonists of collagen-induced platelet aggregation and adhesion have been found in salivary glands of blood feeders. Here we report the first collagen-binding protein from salivary glands of a black fly. This molecule prevents thrombosis in mice without causing significant bleeding, making it an attractive candidate as an antithrombotic agent. Because blackflies and mosquitoes shared a common blood feeding ancestor approximately 250 million years ago, it appears that collagen-binding activity in salivary glands was an evolutionary innovation present in an ancient dipteran ancestor. Our work highlights the central role of inhibition of platelet aggregation as a vital salivary function in blood feeding arthropods.

## Introduction

Salivary glands (SGs) of blood feeding arthropods have been studied for their roles in blood feeding and pathogen transmission to vertebrate hosts. As in other bloodsucking Nematocera, black flies require a blood meal for egg development. To acquire a blood meal, the mandibles of the fly cut into the skin with rapid scissor-like movements, causing blood to pool from which it will feed, with blood feeding usually taking four to five minutes [1]. This feeding behavior triggers the hemostatic response of the vertebrate host against blood loss, which represents a formidable barrier to efficient blood feeding [2].

The first step in the hemostatic cascade is platelet interaction with the exposed extracellular matrix at sites of injury. Collagen is recognized as the most thrombogenic component of the subendothelial matrix. Endothelial damage—such as that caused by blood feeding arthropods—can lead to exposure of collagen to circulating blood, in particular to platelets, leading to thrombogenesis. Multiple collagen receptors have been identified on the platelet surface including immunoglobulin superfamily member GPVI, GPIb and integrin α_2_β_1_, among others (reviewed in [3]). These individual receptors likely play specific roles to mediate collagen-induced platelet adhesion, activation, and consolidation [3], [4]. Absence of any of these components can lead to serious physiologic consequences. For example, von Willebrand disease caused by quantitative or qualitative defects of vWF can cause excessive mucocutaneous bleeding after even minor tissue damage [5].

To counteract the hemostatic system of the host, saliva of blood feeding arthropods contains a complex array of pharmacologically active compounds that act as anticlotting, antiplatelet, vasodilator, anti-inflammatory, and immunomodulatory compounds. Some functional and biochemical characterizations from black fly SGs have previously been reported [6]–[9]. Among the salivary platelet aggregation inhibitors in mosquitoes, it was recently discovered that *Anopheles stephensi* (AAPP) and *Aedes aegypti* (Aegyptin) express a collagen-binding protein that inhibits collagen-induced platelet aggregation by blocking its interaction with three major ligands, namely, GPVI, von Willebrand factor (vWF), and integrin α_2_β_1_ [10]–[12]. These mosquito proteins have a low complexity and acidic amino terminus region rich in glycine/aspartate/glutamate and a relatively more conserved and complex carboxyterminus. Proteins with these characteristics were found in black flies [13]–[15]; however, their overall identity was only 25% when aligned to mosquito proteins [2]. Black flies and mosquitoes share a common blood feeding ancestor at ∼250 million years ago (MYA) [16], giving ample time for diversification of this protein family, although the biophysical, biochemical, and pharmacologic characterization of this protein family in black flies remains to be elucidated. To the extent that they are similar to those of mosquitoes, a point could be made for their orthologous relationship, despite accelerated evolution, probably driven by their hosts' immune pressure over millions of years [17].

Here we report the first collagen-induced platelet aggregation inhibitor from *Simulium nigrimanum* SGs (*Simulium* platelet aggregation inhibitor, Simplagrin). Simplagrin specifically inhibits vWF interaction with collagen under static conditions and completely blocks platelet adhesion to collagen under flow conditions at high shear rates. Simplagrin binds to the vWF-recognition peptide (RGQOGVMGF) with an affinity (K_D_ 11.1±0.59 nM) similar to that of Simplagrin collagen I and III (5.6±0.52 nM and 2.1±0.35 nM, respectively). Furthermore, Simplagrin prevents laser-induced carotid thrombus formation in mice *in vivo* without significant bleeding. From an evolutionary viewpoint, our results support the orthology of the Aegyptin clade in bloodsucking Nematocera and the hypothesis of a faster evolutionary rate of salivary function of distantly related proteins, and the central role of platelet aggregation inhibition in blood feeding arthropods.

## Methods

### Reagents

Adenosine diphosphate (ADP) and phorbol myristate acetate were obtained from Sigma (St. Louis, MO, USA). Ristocetin and arachidonic acid (Ara) were from Chrono-Log Corp. (Haverton, PA, USA). 9,11 dideoxy 9α,11α methanoepoxy prostaglandin F2α (U46619) was purchased from Cayman Chemicals (Ann Arbor, MI, USA); thrombin receptor-activating peptide (TRAP) was from EMD Biosciences (La Jolla, CA, USA). Thrombin and PPACK (D-phenylalanyl-L-propyl-L-arginine chloromethyl ketone) were from Haematologic Technologies (Essex Junction, VT, USA). Convulxin was from Santa Cruz Biotechnology, Inc, (Santa Cruz, CA, USA) and GPVI-His from R&D Systems (Minneapolis, MN, USA). Soluble human collagen type I, III, IV, V and VI were also from R&D Systems. Synthesis and preparation of collagen derived peptides were as described in [11].

### Ethics statement

Public Health Service Animal Welfare Assurance #A4149-01 guidelines were followed according to the NIAID and the National Heart, Lung, and Blood Institute (NIH) Office of Animal Care and Use (OACU). These studies were carried out according to the NIAID and NHLBI animal study protocol (ASP) approved by the OACU Committee, with approval IDs ASP-LMVR3 and ASP-2-CB-38(R) Amendment 18.

### Animals

Adult female mice weighing 15–25 g were housed under controlled conditions of temperature (24±1°C) and light (12 hours light starting at 7:00 am), and all experiments were conducted in accordance with standards of animal care defined by the Institutional Committees (NIH).

### Expression, purification, and labeling of recombinant Simplagrin

Simplagrin expression construct was based on Sim-50 sequence (ACZ28269) and engineered to contain the mature protein and a 6X-His tag before the stop codon. The synthetic, codon optimized gene was produced by BioBasic Inc. (Markham, ON, Canada) and subcloned into VR2001 TOPO vector (modified version of the VR1020 vector; Vical Incorporated [San Diego, CA, USA]). About 1 mg of plasmid DNA (VR2001-Simplagrin construct) was obtained using GeneElute HP endotoxin free plasmid MEGA prep kit (Sigma). The plasmid was purified through a 0.22 µm filter, and the recombinant protein was produced by transfecting FreeStyle293 F cells (Invitrogen, San Diego, CA, USA). After 72 hours, transfected cell culture was harvested and the supernatant containing the secreted recombinant protein was centrifuged (100×*g*, 15 minutes), frozen, and shipped to our lab until purification.

HEK293 cell supernatant containing the recombinant protein was loaded onto a Ni^2+^ column (5 mL bed volume; GE Healthcare, Piscataway, NJ, USA) following the manufacturer's directions. Fractions were step eluted with 5, 20, 300 and 1000 mM imidazole (in 10 mM Tris, 500 mM NaCl, pH 8.0) and then loaded onto a size exclusion column (Superdex 200 HR10/30; GE Healthcare) using the AKTA purifier system. Proteins were eluted isocratically at a flow rate of 0.5 mL/minute in 25 mM Tris, 150 mM NaCl, pH 8.0. Purified recombinant protein was submitted to automated Edman degradation for N-terminal sequencing. To detect purity of Simplagrin, 10 µg of purified protein was loaded in a 4–12% NuPAGE gel (Life Technologies, Gaithersburg, MD, USA) and the gel stained with Coomassie blue. FITC labeling of Simplagrin was carried out using the FITC protein labeling kit according to the manufacture's recommendations (Life Technologies). Briefly, labeling reaction was incubated at room temperature (RT) for two hours, and unreacted dye was removed by size exclusion chromatography as described above. The final concentration and degree of labeling were determined by measuring the absorbance spectrum of the labeled Simplagrin as indicated below.

### Simplagrin determinations

Concentration of purified Simplagrin was estimated by its absorbance at 280 nm using an ND1000 spectrophotometer (NanoDrop Technologies, Wilmington, DE, USA) and corrected according to the calculated molar extinction coefficient ε_280 nm_ of 10,930 M^−1^cm^−1^.

### Dynamic light scattering plot

The purity and solution state of purified Simplagrin were analyzed using size exclusion chromatography with online multiangle light scattering (SEC-MALS-QELS-HPLC), refractive index, and ultraviolet (UV) detection. The instrument, a Waters Corporation (Milford, MA, USA) HPLC model 2695 and photodiode array detector model 2996 operated by Waters Corporation Empower software, connected in series to a Dawn EOS light scattering detector and Optilab DSP refractive index detector (Wyatt Technology, Santa Barbara, CA, USA), was used as directed by the manufacturer. Wyatt Technology's Astra V software suite was used for data analysis and processing. For separation, a TSK gel G3000PWxl column (7.8 mm×30 cm; 6 µm particle size) (Tosoh Bioscience, King of Prussia, PA, USA) was used with a TSK gel Guard PWxl column (6.0 mm×4.0 cm, 12 µm particle size). The column was equilibrated in mobile phase (1.04 mM KH_2_PO_4_, 2.97 mM Na_2_HPO_4_ • 7H_2_O, 308 mM NaCl, 0.5 M urea, pH 7.4, 0.02% sodium azide) for at least 60 minutes at 0.5 mL/min prior to sample injection. SEC-MALS-HPLC analysis was performed on the Simplagrin using an isocratic elution at 0.5 mL/min in mobile phase. Bio-Rad gel filtration standards were run for size comparisons.

### Enzymatic deglycosylation of Simplagrin

To evaluate whether recombinant Simplagrin underwent glycosylation by HEK293 cells, an enzymatic deglycosylation assay was carried out. Fifteen µg of Simplagrin or Lundep (positive control [18]) were denatured at 95°C for 5 minutes and then treated with a deglycosylase mix containing PNGase F, sialidase, β-galactosidase, glucosaminidase, and O-glycosidase (Enzymatic DeGlycoMx Kit; QA-Bio, Palm Desert, CA, USA). After three hours of enzymatic deglycosylation, the samples were submitted to electrophoresis. Electrophoretic mobility of Simplagrin before and after enzymatic deglycosylation was compared in a Coomassie blue stained NuPAGE (Life Technologies).

### Circular dichroism (CD) of Simplagrin

CD spectra were measured by a Jasco J-715 spectropolarimeter with the solutions in a 0.1-cm path length quartz cuvette in a cell holder thermostated by a Neslab RTE-111 circulating water bath. Spectra were scanned four times from 260 to 200 nm and averaged (speed 50 nm/min, time constant one second) at 25°C. After baseline correction, CD spectra were converted into mean residue ellipticity values using the formula described in [11].

### Surface plasmon resonance (SPR) analysis

All SPR experiments were carried out in a T100 instrument (GE Healthcare) following the manufacturer's instructions. Sensor CM5, amine coupling reagents, and buffers were also purchased from GE Healthcare. HBS-P (10 mM Hepes, pH 7.4, 150 mM NaCl, and 0.005% (v/v) P20 surfactant was used as the running buffer for all SPR experiments. All SPR experiments were analyzed using the Biacore Evaluation software v2.0.3 provided by GE Healthcare. All SPR experiments were carried out three times.

### Immobilization and kinetic analysis. Collagen-Simplagrin and RGQPGVMGF-Simplagrin interactions

Collagen type I or type III (20 µg/mL) in acetate buffer (pH 4.5) was immobilized over a CM5 sensor via amine coupling. The immobilization target was aimed to 800 resonance units (RU), resulting in a final immobilization of 796.6 RU for collagen type I and 818.6 RU for collagen type III. Blank flow cells were used to subtract the buffer effect on sensograms. Alternatively, Simplagrin (50 µg/mL in acetate buffer; pH 4.5) was immobilized on a CM5 at a surface density of 500 RU. Kinetic experiments were carried out with a contact time of 180 seconds at a flow rate of 30 µL/minute at 25°C. Simplagrin-collagen complex dissociation was monitored for 1,800 seconds, and the sensor surface was regenerated by a pulse of 20 seconds of 10 mM HCl at 40 µL/minute. Sensograms were fitted using the 1∶1 Langmuir interaction model. For steady-state affinity calculations, Simplagrin was immobilized as described above and different concentrations of soluble collagen (type I–VI) were used as analyte. Steady-state affinity was calculated by plotting the equilibrium response (Req) levels against the analyte concentration. The affinity constant (K_D_) is reported as the analyte concentration at 50% of saturation.

### Thermodynamic analysis

Thermodynamic parameters for Simplagrin collagen type I and III interaction were obtained from independent kinetic experiments using the Thermo Wizard assay program. Briefly, six different concentrations of recombinant Simplagrin (10, 25, 50, 100, 250, and 500 nM) were injected over immobilized collagen type I or III at 15, 20, 25, 30, 35, and 40°C. Contact time, dissociation time, and regeneration of the sensor surface were done as described above. Resulting sensograms were fitted to the 1∶1 interaction model with global Rmax. The association (Ka) and dissociation (Kd) rate constants, as well as the affinity constant (K_D_), were obtained and fitted to a linear form of the van't Hoff equation to estimate the ΔH and ΔS.

### Identification of collagen-binding activity of Simplagrin

This experiment was essentially carried out as described by Calvo et al. [10]. Briefly, Simplagrin was immobilized on a CM5 sensor chip (GE Healthcare), and different analytes were injected over the sensor for 120 seconds at a flow rate of 20 µL/minute. Complex dissociation was monitored for 400 seconds, and the sensor chip surface was regenerated with a 10 second pulse of 10 mM HCl at 30 µL/minute.

### Solid-phase analysis of Simplagrin-collagen interaction

Human soluble collagen type I, III, IV, V, or rat tail type I (25 µg/mL in PBS; pH 7.4) were immobilized overnight at 4°C. Wells were washed with PBS and blocked with BSA (2% v/v, in PBS) for two hours. Simplagrin (0–3 µM) diluted in PBS-T (PBS, 1% BSA, 0.05% Tween, pH 7.4) was added in quadruplicates. After two hours, wells were washed in PBS-T and incubated with anti-Simplagrin (1 µg/mL) in the same buffer. After one hour, wells were washed 3× and incubated with alkaline phosphatase-coupled anti-rabbit IgG (1∶5000, in PBS-T). After one hour incubation at 37°C, the wells were washed four times with PBS-T and stabilized p-nitrophenyl phosphate liquid substrate (Sigma) was added. Colorimetric analysis was performed by measuring absorbance values at 405 nm.

### Platelet aggregation

Platelet aggregation was measured as described previously using an aggregometer [10]. Briefly, platelet rich plasma (PRP) was prepared from medication free donors by plateletpheresis (Department of Transfusion Medicine, NIH Clinical Center). Diluted PRP (1∶3 in Tyrode's buffer) in the presence or absence of Simplagrin (10 µL) or PBS (control) were pre stirred in the aggregometer for three minutes to monitor preaggregation effects. Aggregation was then induced using the following agonists: fibrillar collagen (1 or 10 µg/mL), phorbol 12-myristate 13-acetate (0.5 µM), ADP (10 µM), TRAP (5 µM), U46619 (0.7 µM), arachidonic acid (1 mM), epinephrine (50 µM), ristocetin (1 mg/mL), convulxin (100 pM), and thrombin (0.1 U/mL). All experiments were carried out in triplicate using PRP from three different healthy donors.

### vWF binding to RGQOGVMGF peptide

Polystyrene plates were coated with 100 µL of collagen type III (0.03 µg/mL), RGQOGVMGF peptide (30 µg/mL), or a 2% (w/v) solution of bovine serum albumin (BSA) diluted in PBS for two hours at 37°C. After washing twice with PBS to remove unbound protein, residual binding sites were blocked by adding 5 mg/mL denatured BSA overnight at 4°C. After washing 3× with 50 mM Tris HCl, 150 mM NaCl, and 0.05% (v/v) Tween 20, pH 7.4 (TBS-T), increasing concentrations of recombinant Simplagrin (ranging from 0.05 to 3 µM) were added to the wells and incubated at 37°C for one hour. Wells were washed again and incubated with 3 nM of vWF factor VIII free (Haematologic Technologies Inc.) in TBS-T supplemented with 2% (w/v) BSA. After one hour at 37°C, wells were washed 3× with TBS-T, and a polyclonal rabbit anti human vWF (DakoCytomation, Glostrup, Denmark) was added (1∶500 in TBS-T) and incubated for one hour at 37°C. After three washes with TBS-T, an alkaline phosphatase conjugate anti-rabbit IgG (whole molecule; Sigma) was added (1∶10,000) and incubated at 37°C for 45 minutes. Before adding stabilized p-nitrophenyl phosphate liquid substrate (Sigma), wells were washed 6× with TBS-T. After 30 minutes of substrate conversion, the reaction was stopped with 3 N NaOH and the absorbance read at 405 nm using a Thermomax microplate reader (Molecular Devices, Sunnyvale, CA, USA). Net specific binding was obtained by subtracting optical density values from wells coated only with BSA from the total binding measured as described above. All experiments were performed in triplicate.

### Polyclonal antibody production and western blot

Polyclonal antibodies against Simplagrin (wild type, full length) were raised in rabbits by Spring Valley Laboratories, Inc. (Woodbine, MD, USA) using a standard protocol. Briefly, rabbits were immunized 3× with 125 µg of Simplagrin every 21 days and the serum collected at day 72. A 10-mL aliquot of rabbit serum (immunized or naïve) was diluted to 50 mL in phosphate buffer, pH 6.5, and loaded onto a 5 mL HiTrap protein A HP column (GE Healthcare) and the IgG eluted with a linear gradient of citric acid (100 mM, pH 3.4) using an Akta purifier system (GE Healthcare). Fractions containing purified IgG were pooled and dialyzed against 1× PBS for 16 hours at 4°C. IgG quantification was based on 1 absorbance unit at 280 nm = 0.7 mg/mL. For western blot analysis, Simplagrin (5 µg) was electrophoresed in a 4–12% NuPAGE in MES buffer (Invitrogen). After electrophoresis, samples were electrotransferred onto nitrocellulose membrane using an iBlot gel transfer system (Invitrogen). The membrane was incubated overnight at 4°C with TBS (25 mM Tris, 150 mM NaCl, pH 7.4) containing 5% (w/v) powdered nonfat milk (blocking buffer), followed by incubation for 90 minutes at RT with purified anti-Simplagrin rabbit IgG diluted 1∶1000 in blocking buffer. The membrane was washed 4× with TBS-T and incubated with goat anti-rabbit alkaline phosphatase conjugated (Sigma) diluted 1∶10,000 in blocking buffer. The immunoblot was developed by addition of 1 mL of Western Blue stabilized substrate for alkaline phosphatase (Promega, Madison, WI, USA).

### In solution competition assays

Experiments were performed to detect whether Simplagrin blocks collagen interaction with GPVI, vWF, or integrin α_2_β_1_. Recombinant GPVI (25 µg/mL) in acetate pH 4.5 buffer was immobilized on a CM5 sensor with a final surface density of 1,753.2 RU. A blank flow cell was used to subtract any effect of buffer in the refractory index change. Then different concentrations (3.175, 6.125, 12.5, 25, and 50 µg/mL) of collagen I alone (control) or previously incubated (15 minutes at RT) with 500 nM of Simplagrin in HBS-P buffer was injected over immobilized GPVI for 120 seconds at 20 µL/minute. Complex dissociation was monitored for 400 seconds. Sensor surface was regenerated between runs by a 30 second pulse of glycine solution, pH 1.5. To verify that immobilized GPVI was still active after all the injection and regeneration cycles, 50 µg/mL of collagen I was injected for 120 seconds at a flow rate of 20 µL/minute and the resulting sensogram compared with the one obtained before. A control experiment was carried out using convulxin at different concentrations (2.5, 5, and 10 nM) incubated with buffer or saturating concentrations of Simplagrin (500 nM) followed by injection of the mixture over immobilized GPVI, as described above. Alternatively, immobilized collagen type III was allowed to interact with Simplagrin alone or preincubated with saturating concentrations of RGQOGVMGF peptide in HBS-P. Contact time, dissociation and regeneration of the sensor chip were carried out as described above.

### Blocking of vWF binding to collagen by Simplagrin

Polystyrene plates were coated with 100 µL of collagen type III (0.3 µg/mL) or a 2% (w/v) solution of BSA diluted in PBS for two hours at 37°C. After washing twice with PBS to remove unbound protein, residual binding sites were blocked by adding 5 mg/mL denatured BSA overnight at 4°C. After washing 3× with 50 mM Tris HCl, 150 mM NaCl, and 0.05% (v/v), TBS-T, pH 7.4, increasing concentrations of recombinant Simplagrin (ranging from 0.0015 to 1.5 µM) were added to the wells and incubated at 37°C for one hour. Wells were washed again and incubated with 3 nM of vWF factor VIII free (Haematologic Technologies Inc.) in TBS-T supplemented with 2% (w/v) BSA. After one hour at 37°C, wells were washed 3× with TBS-T, and a polyclonal rabbit anti-human vWF (DakoCytomation) was added (1∶500 in TBS-T) and incubated for one hour at 37°C. After three washes with TBS-T, an alkaline phosphatase conjugate anti-rabbit IgG (whole molecule; Sigma) was added (1∶10000) and incubated at 37°C for 45 minutes. Before adding the stabilized p-nitrophenyl phosphate liquid substrate (Sigma), wells were washed 6× with TBS-T. After 30 minutes of substrate conversion, the reaction was stopped with 3 N NaOH and absorbance read at 405 nm using a Thermomax microplate reader (Molecular Devices). Wells coated only with BSA were used as assay blanks. All experiments were performed in triplicate.

### Platelet adhesion under high-shear stress

Coverslips (22×22 mm, no. 0) were treated with H_2_SO_4_: H_2_O_2_ (4∶1) for 20 minutes to remove contaminants, followed by ultrasonic washing with deionized water and ultraviolet cleaning. Coverslips were coated with fibrillar (100 µg/mL; Chronolog) or soluble collagen type III (100 µg/mL) for ten minutes, rinsed in deionized water, and incubated overnight with denaturated BSA (4 mg/mL). Coverslips were treated with 150 µL of Simplagrin (0–5 µM) for 15 minutes and excess removed by inversion. A coated coverslip formed the bottom of the parallel plate flow chamber (Glycotech), and a silicone rubber gasket determined the flow path height of 254 µm. Anticoagulated blood (50 µM PPACK) was mixed with Simplagrin and aspirated using an infusion/withdrawal pump (Model 940; Harvard Apparatus) through the flow chamber at a flow rate of 0.6 mL/minute, producing a shear rate of 1,500 s^−1^ as described before [10]. Blood was perfused for 240 seconds, followed by washing with Tyrode's buffer to remove unbound platelets and red blood cells. Platelet adhesion under flow conditions was recorded using a Leica DMI6000 microscope (Leica Microsystems, Inc., Bannockburn, IL, USA) using 100x objective with NA = 1.30, and an ORCA ER digital camera (Hamamatsu Photonic Systems, Bridgewater, NJ, USA). Image acquisition and the digital camera were controlled by ImagePro 5.1 software (Media Cybernetics, Silver Spring, MD, USA).

### Photochemically induced carotid artery thrombosis in mice

Female mice (C57BL/6, 20–25 g weight) were anesthetized with intramuscular xylazin (16 mg/kg) followed by ketamine (100 mg/kg). The right common carotid artery was isolated through a midline cervical incision, and blood flow was continuously monitored using a PRB Doppler flow probe coupled to a TS420 flow meter (Transonic Systems, Ithaca, NY, USA). Fifteen minutes before induction of thrombosis, animals were injected in the cava vein with Simplagrin (20 or 100 µg/kg) or PBS (control). Thrombosis was induced by slow injection (over 2 minutes) of 90 mg/kg body weight of rose Bengal dye (Fisher Scientific, Pittsburgh, PA, USA) into the cava vein at a concentration of 60 mg/mL. Just before injection, a 1.5 milliwatt (mW), 540 nm green-light laser (Melles Griot, Carlsbad, CA, USA) was applied to the desired site of injury from a distance of 3 cm. Mean carotid artery blood flow was monitored for 80 minutes or until stable occlusion occurred, at which time the experiment was terminated.

### Histologic analysis of damaged arteries

Following *in vivo* thrombus formation measurements in mice, injured carotid arteries were excised and fixed in 2.5% glutaraldehyde in 0.1 M cacodylate buffer, pH 7.2, and shipped to Histoserv Inc. (Germantown, MD, USA) for histologic sectioning and staining with hematoxylin and eosin. Samples were examined by light microscopy in a Zeiss-LSM 510 microscope.

### Tail bleeding

BALB/c mice (female, 15–17 g body weight) were anesthetized as above with a combination of xylazine and ketamine (16 and 100 mg/kg, respectively) and Simplagrin (20 or 100 µg/kg) or PBS in a final volume of 100 µL were administered intravenously via tail vein. After 15 minutes, the mouse tail was cut within 2 mm of diameter and carefully immersed in 40 mL distilled water at RT. The hemoglobin content in water solution (absorbance at 540 nm) was used to evaluate blood loss.

### Statistical analysis

Results are expressed as means ± SEM. Statistical significance was determined using Student's *t*-test or analysis of variance (Bonferroni post-test comparison) using GraphPad v6.0 (GraphPad Software Inc., San Diego, CA, USA). Significance was set at P<0.05.

## Results

### Initial observations

**Figure 1:**
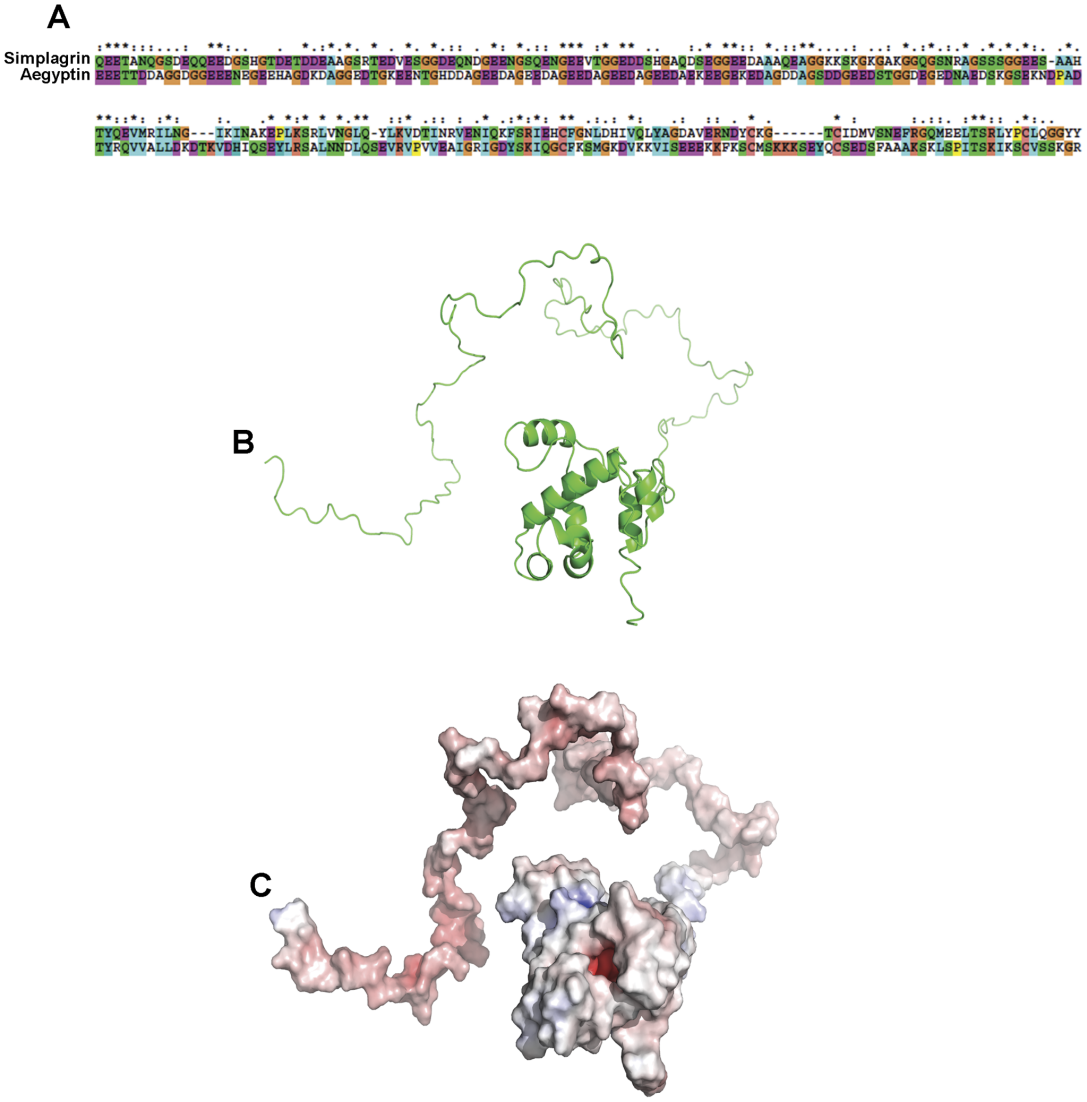
In silico analysis of Simplagrin. Transcriptome analysis of female salivary glands from the black fly *S. nigrimanum* revealed the presence of an abundant transcript distantly related to Aegyptin, a salivary collagen binding protein from *Aedes aegypti.* (A) Amino acid alignment of mature Simplagrin with Aegyptin reveals low overall identity (25%) between both proteins. No putative conserved domain was found. (B) Three-dimensional structure prediction of Simplagrin showing a ribbon diagram of the model generated using PyMOL. Coordinates were generated automatically by I-TASSER software. (C) Surface map of Simplagrin generated by PyMOL APBS tools. Electrostatic potential surfaces of the model showing the positively (blue) and negatively (red) charged surfaces.

Previous black fly sialotranscriptome analysis [14] revealed the presence of expressed sequence tags (ESTs) distantly related to Aegyptin, a mosquito platelet aggregation inhibitor [10]. Cluster Sim-50 (Simplagrin) is the fourth most abundant transcript in the SGs of *S. nigrimanum* and accounts for approximately 3% of all sequenced ESTs (36 of 1215). The cDNA of Simplagrin has an open reading frame of 861 bp coding for a protein of 286 amino acids (aa). It has a predicted signal peptide [19] of 20 aa, indicative of secretion. The predicted mature Simplagrin has a calculated molecular mass of 28,828.88 Da, with an isoelectric point of 4.02 with six potential N-glycosylation sites. Alignment of Simplagrin with Aegyptin (Figure 1A) shows an overall amino acid identity of 25%. Although having small amino acid conservation, these black fly proteins recognize mosquito proteins including Aegyptin by psiblast, and have a non-promiscuous motif G-x(27,30)-L-x-S-x(5)-L-Q-x(16,17)-S-x-I-x(2)-C-F-x(20)-C-x(3,9)-C at their carboxyterminus that is common to mosquito and black fly proteins. A more detailed phylogenetic analysis of Aegyptin-like proteins from Nematocera shows robust clades for *Anopheline*, *Culicine*, *Simulium*, and *Phlebotomus* [2]. Three-dimensional modeling of Simplagrin shows that this protein has a low complexity/disorganized aminoterminal region with a relatively more complex carboxyterminus domain (Figure 1B and C).

### Expression and purification of recombinant Simplagrin: biochemical and biophysical characterization

**Figure 2:**
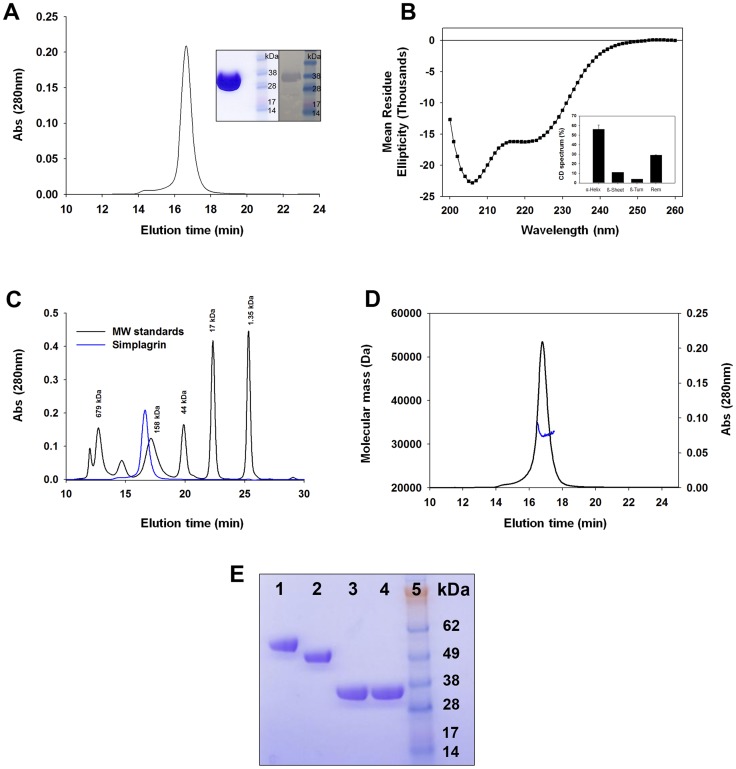
Expression of recombinant Simplagrin and biophysical analysis. (A) Recombinant Simplagrin was expressed as a secreted protein in HEK293 cells. Supernatant containing Simplagrin was concentrated and loaded onto a Ni^+2^-Hitrap column for affinity purification and further purified to homogeneity by size exclusion chromatography. Inset: Coomassie stained NuPAGE and western blot using rabbit anti-Simplagrin antibodies. (B) Circular dichroism (CD) spectroscopy analysis of Simplagrin shows that it mainly comprises α-helix (59%) followed by unordered/disorganized (29%) secondary structures. Inset shows the calculated percentages of secondary structures determined by CD analysis. (C) Analytical size exclusion chromatography shows that Simplagrin runs at a higher than expected molecular weight. (D) Hydrodynamic property of Simplagrin demonstrates its monomeric, elongated form with a hydrodynamic radius of 5.6 nm. The calculated molecular weight of Simplagrin by dynamic scattering plot was 32 kDa (blue line in the chromatogram). (E) Recombinant Simplagrin is not glycosylated in HEK293 cells. Evaluation of putative glycosylation of recombinant Simplagrin was evaluated using DeGlycoMx kit. Fifteen µg of Simplagrin or Lundep (positive control) were heat denatured and treated with an enzymatic deglycosylase mix. After three hours at 37°C, samples were electrophoresed in a NuPAGE-MES and stained with Coomassie blue. Lane 1: Lundep, 2: Lundep+DeGlycoMx, 3: Simplagrin, 4: Simplagrin+DeGlycoMx, 5: mW standard (SeeBlue Plus2 in kDa).

Recombinant Simplagrin was expressed in HEK293 cells and purified by affinity and size exclusion chromatography (Figure 2A) as described by Calvo et al. [10]. Identity and purity of the recombinant protein was verified by N-terminal sequencing and liquid chromatography-mass spectrometry analysis (not shown). Protein A-purified polyclonal antibodies raised against Simplagrin in rabbits recognized the recombinant protein in western blot (Figure 2A, inset). Circular dichroism spectroscopy (CDS) analysis of Simplagrin shows that it comprises primarily an α-helix (56%) followed by 29% of remainder/disorganized, 15% of β-sheet, and 4% β-turn structures (Figure 2B). This distribution of secondary structures is in agreement to the modeled 3D structure of Simplagrin (Figure 1B and C).

Although the predicted molecular mass of Simplagrin is 30.25 kDa (including the 6xHis-tag), it is eluted at a higher apparent molecular mass of 160 kDa when loaded on a size-exclusion column (Figure 2C). This abnormal chromatographic pattern suggests that Simplagrin could be oligomeric or non-globular in nature. To further investigate this feature, we used dynamic light-scatter plotting to analyze the hydrodynamic radius of Simplagrin. Our results show that Simplagrin has an elongated monomeric form with a hydrodynamic radius of 5.6 nm and a calculated mass of 32 kDa (Figure 2D). This difference between the expected and calculated molecular mass of 30.25 kDa may be due to incomplete separation of the aggregate peak (7%) and the monomer peak (93%). No glycosylation was found when Simplagrin was treated with an enzymatic deglycosylase mix (Figure 2E). A similar result was reported for recombinant Aegyptin [10].

### Identification of collagen as the molecular interaction partner of Simplagrin

**Figure 3:**
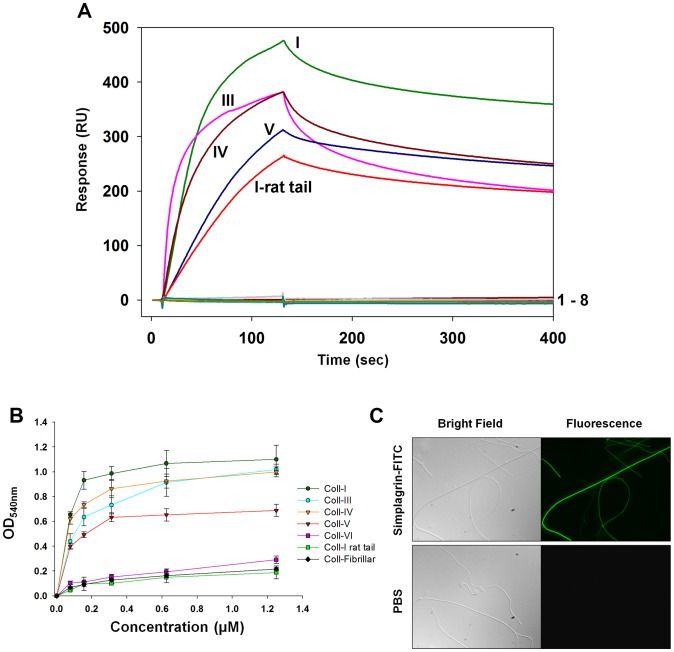
Simplagrin specifically binds to collagen. (A) Surface plasmon resonance: initial screening over immobilized Simplagrin, showing it exclusively binds to collagen (human type I, III, IV, V, and type I from rat tail). No detectable binding was observed to (1–8) coagulation factors IIa, Va and Xa, vitronectin, laminin, fibronectin, vWf, and fibrinogen. (B) Solid-phase assay shows that Simplagrin binds to immobilized collagens on 96 well plates. Binding was detected by ELISA using rabbit anti-Simplagrin antibodies. (C) Visualization of Simplagrin collagen interaction. Fluorescent microscopy showing direct binding of FITC labeled Simplagrin on coverslip coated with fibrillar collagen. Collagen coated coverslips were incubated with 1 µM of FITC labeled Simplagrin for 15 minutes and then washed 5 times with PBS before mounting. Collagen fibrils revealed by bright-field and fluorescence shows that it lacks of autofluorescence.

Because Simplagrin appears to belong to the Aegyptin family of salivary proteins, we carried out a collagen-binding assay using SPR, solid-phase experiments, and fluorescence microscopy. For SPR experiments, Simplagrin was immobilized on a CM5 sensor chip and different analytes were flowed over the sensor's surface. Despite the low similarity between Aegyptin and Simplagrin at the amino acid level, SPR analysis identified collagen (human types I, III, IV, V, and VI, and type I from rat tail) as the molecular partner of Simplagrin (Figure 3A). No detectable binding was observed to vitronectin, fibronectin, fibrinogen, vWF, recombinant platelet receptors GPVI and integrin α_2_β_1_, and coagulation factors IIa and Xa (Figure 3A). No effect of Simplagrin on blood clotting, vasodilation, inflammation, or murine splenocyte proliferation was found (not shown). We also verified the collagen binding activity of Simplagrin using a solid phase. For this experiment, collagen-coated wells were incubated with different concentrations of Simplagrin and the binding activity measure by an ELISA based assay utilizing anti-Simplagrin antibodies. Figure 3B shows results for the solid phase assay similar to those found with SPR analysis. Alternatively, FITC labeled Simplagrin was used to visualize its binding to fibrillar collagen. For this experiment, FITC labeled Simplagrin (1 µM) was allowed to interact with fibrillar collagen (immobilized on a glass coverslip). After 15 minutes of incubation at RT, the coverslips were washed five times with TBS and analyzed under bright field and fluorescence microscope. Figure 3C (upper panel) shows that FITC labeled Simplagrin binds to the collagen fibrils immobilized on the cover slip. Collagen incubated with buffer alone did not show autofluorescence under the same conditions (Figure 3C, lower panel).

### Kinetic and thermodynamic analysis of Simplagrin-collagen interaction

**Figure 4:**
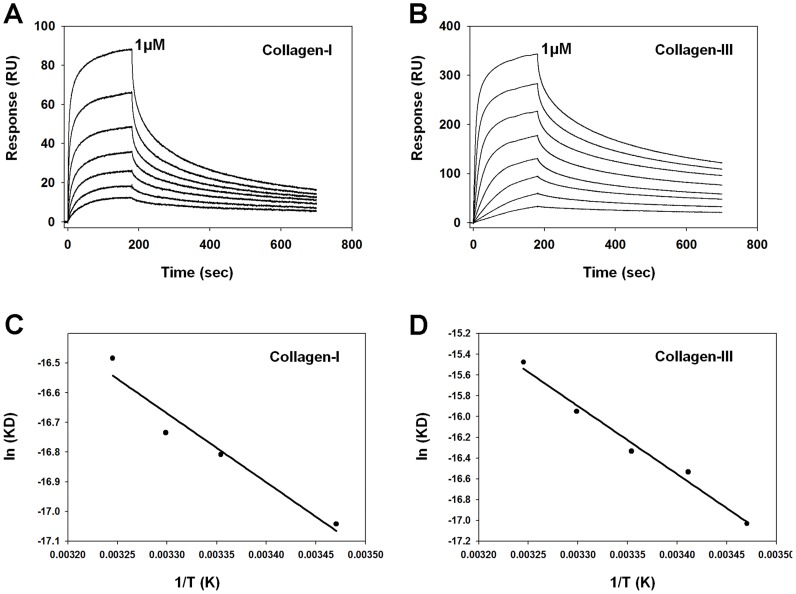
Kinetic and thermodynamic analysis of Simplagrin collagen interaction. (A) and (B) Simplagrin at different concentrations was flowed over immobilized collagen for 180 seconds at 30 µL/mL and the collagen Simplagrin dissociation monitored for 600 seconds. The response data were fitted to a 1∶1 interaction model using global analysis. Simplagrin displays high affinity for collagen type I and III. (C) and (D) Thermodynamic parameters of Simplagrin collagen (I and III) were obtained from independent kinetic experiments using surface plasmon resonance (SPR). Δ*H* and Δ*S* were obtained using the van't Hoff equation. All SPR experiments were carried out in triplicate. Results are representative of typical sensograms. Molar concentrations of collagen were calculated assuming an MW of 250 kDa.

**Figure 5:**
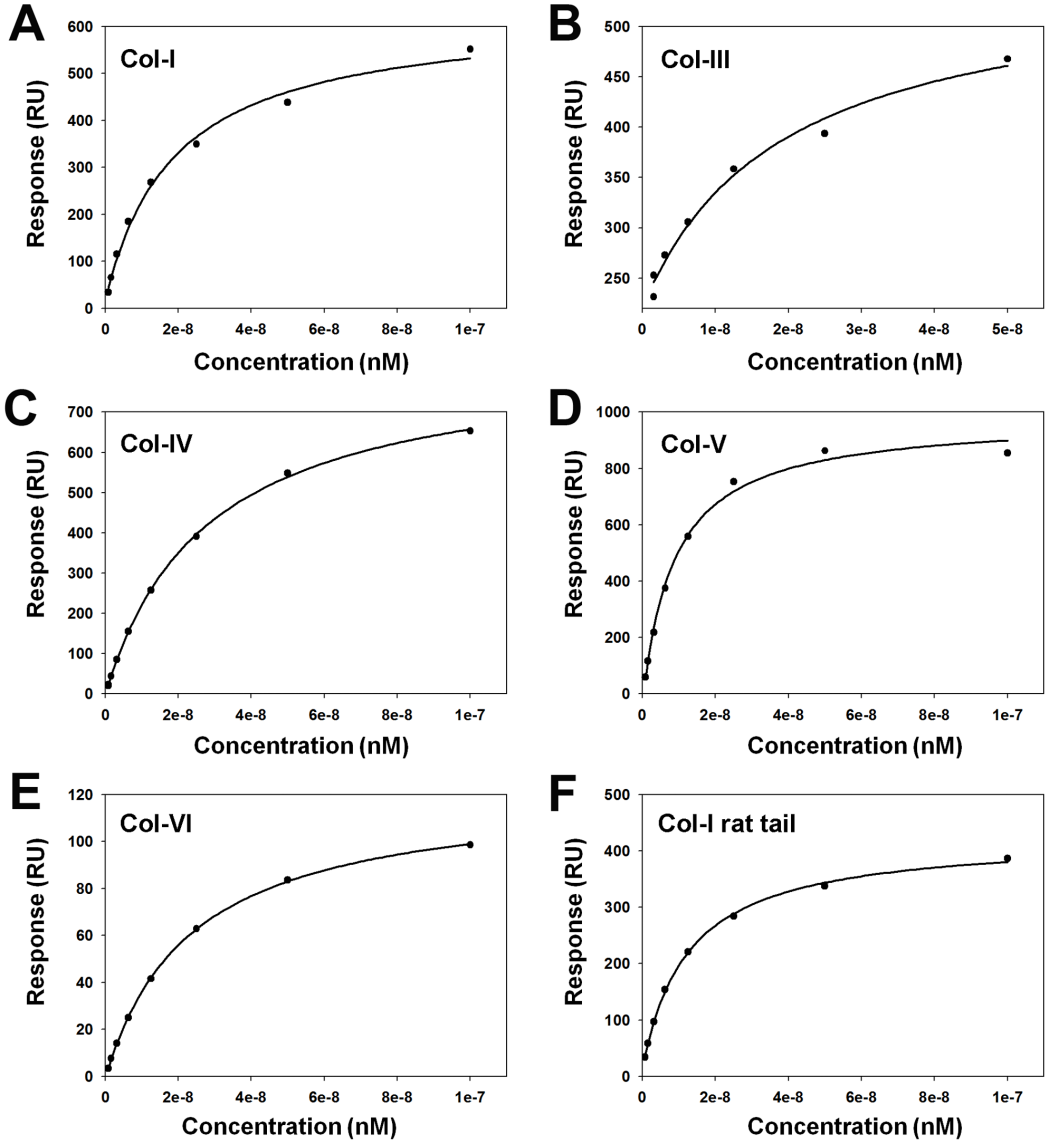
Affinity values for Simplagrin with different collagen types. (A-F) Soluble human collagen type I, III, IV, V, VI and rat tail I were flowed over immobilized Simplagrin. Fitting of steady-state responses from Simplagrin collagen interaction measured by surface plasmon resonance. The steady-state signal reached at the end of the analyte injection (240 seconds at 30 µL/minute) was plotted against the analyte concentration and the resulting curve fitted with a Langmuir 1∶1 binding model.

Due to the collagen binding activity displayed by Simplagrin, a more detailed analysis of Simplagrin-collagen interaction was carried out to calculate the kinetic and thermodynamic constants of this interaction. Immobilized collagen type I and III were used as a ligand, and Simplagrin at concentrations ranging from 0.015–1 µM (serial dilutions) was flowed over the sensor for 180 seconds. Simplagrin-collagen complex dissociation was monitored for 30 minutes, and the resulting sensograms were fitted using a 1∶1 binding model. Sensograms of a typical kinetic experiment are shown in Figure 4A and B. Using SPR, we calculated the K_D_ values for Simplagrin collagen I and III, of 5.51±0.52 nM and 2.10±0.35 nM, respectively (Table 1). Affinity of Simplagrin for human collagen type I, III, IV, V, VI and type I of rat tail was also calculated using Simplagrin as ligand, and the resulting sensograms fitted with a steady-state model (Figure 5). The K_D_ values for these collagens are in the same order of magnitude as those of Simplagrin collagen I and III (Table 2).

**Table 1:** Surface plasmon resonance analysis of Simplagrin-collagen interaction.

Collagen type	ka (1/Ms)	kd (1/s)	K_D_ (nM)
Collagen-I	4.095±0.97E+5	2.137±0.33E-4	5.605±0.052
Collagen-III	1.478±0.12E+5	3.101±0.53E-4	2.102±0.350

Kinetic and binding constants were calculated using a 1:1 interaction binding model. Experiments were carried out in triplicate.

**Table 2:** Binding constant of Simplagrin with different types of collagen.

Collagen type	K_D_ (nM)
Collagen-I	10.34
Collagen-III	9.717
Collagen-IV	27.57
Collagen-V	10.85
Collagen-VI	23.26
Collagen-I (rat tail)	12.39

The steady-state affinity constant was calculated using an equilibrium binding model.

**Figure 6:**
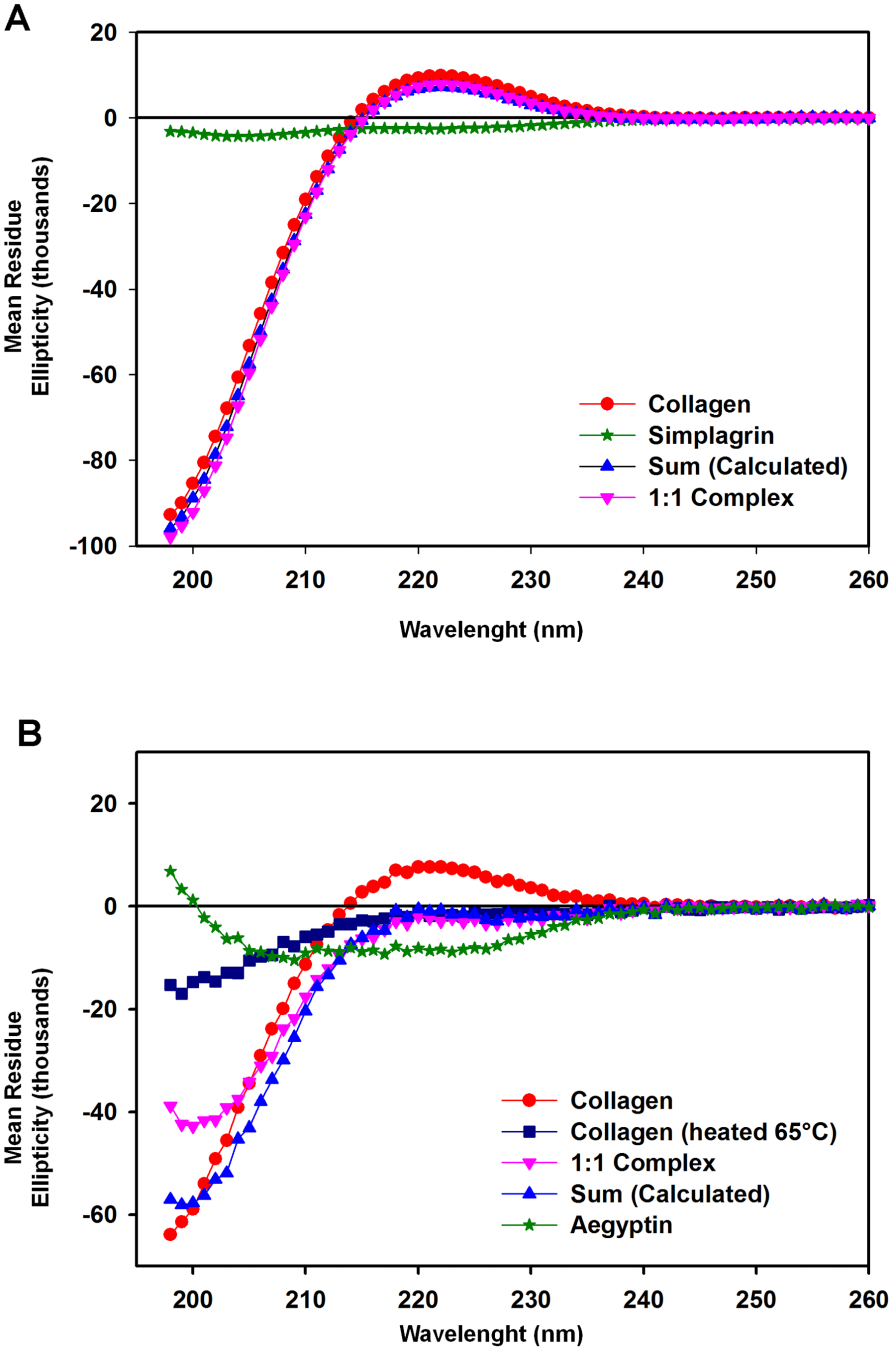
Simplagrin does not induce any significant conformational change upon binding to collagen type I. To verify whether any major conformational change occurs upon collagen Simplagrin interaction, a circular dichroism (CD) spectroscopy of the complex was analyzed. (A) Collagen with or without equal molar concentration of Simplagrin showing no significant conformational change in the 1∶1 complex. (B) CD spectrum of collagen type I in equimolar concentration of Aegyptin showing the structural changes of the collagen molecules, resembling the CD spectrum of heat denatured soluble collagen type I. The CD analysis suggests that a conformational change of the collagen molecule, most likely due to reduction in poly proline II structure, results in a significant decrease of ellipticity of the collagen molecule. This unwinding of collagen may result in loss of collagen interaction with its physiological ligands. All experiments were carried out at 25°C in TBS with 6 µM concentration of collagen and Simplagrin or Aegyptin. Molar concentration of collagen was calculated assuming an average MW of 250 kDa. Sum represents the mathematic sum of measured CD spectra of individual molecules.

For thermodynamic analysis of Simplagrin-collagen interaction, different concentrations of Simplagrin ranging from 25 nM to 500 nM were injected over immobilized collagen type I or III at different temperatures (15, 20, 25, 30, 35, and 40°C). The association (k_a_) and dissociation (k_d_) rate constants, as well as the affinity constant (K_D_), were obtained and fitted to a linear form of the van't Hoff equation to estimate the ΔH, ΔS, and ΔG (Figure 4C and D). Thermodynamic analysis of the Simplagrin-collagen interaction shows that this interaction is both entropically and enthalpically driven with a larger negative ΔG, indicating that this interaction is favorable and occurs spontaneously (3). CDS analysis of Simplagrin-collagen interaction shows that no conformation change occurs during complex formation (Figure 6).

**Table 3:** Thermodynamic analysis of Simplagrin-collagen interaction measured by surface plasmon resonance.

Collagen type	ΔH [kJ/mol]	ΔS [J/(K*mol)]	ΔG [kJ/mol]
Collagen-I	–19±3.3	75±1.1	–42
Collagen-III	–54±4.1	–47±14.0	–40

Thermodynamic parameters were calculated with the van’t Hoff equation.

### Simplagrin inhibits collagen induced platelet aggregation at low collagen concentration

**Figure 7:**
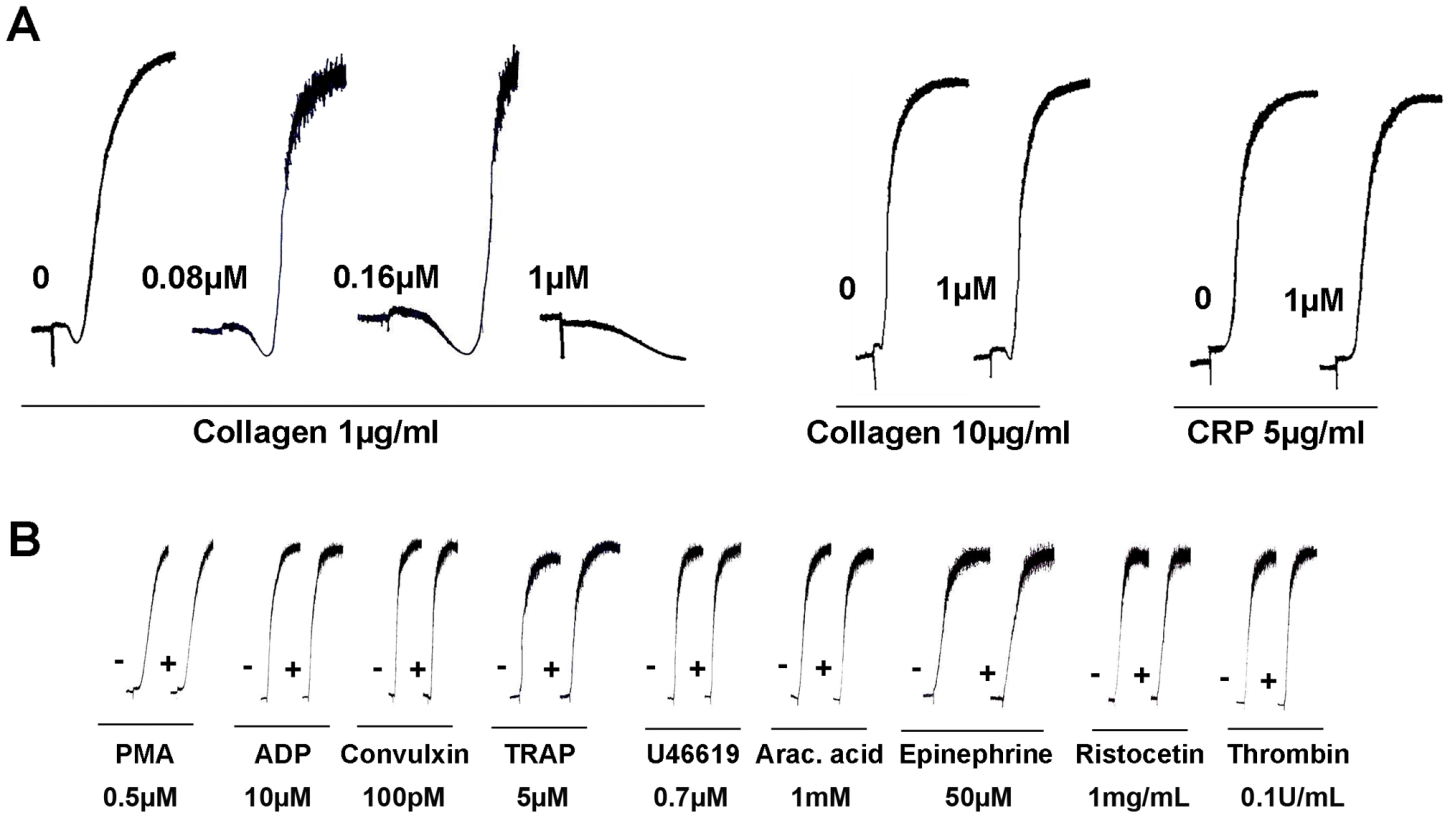
Effect of Simplagrin on platelet aggregation. (A) Human platelet rich plasma from healthy donors was incubated with Simplagrin at different concentrations for two minutes followed by addition of platelet agonists as described under Methods. Platelet aggregation was estimated by turbidimetry under stirring conditions at 37°C. No effect on collagen induced platelet aggregation was observed at 10 µg/mL of collagen or 5 µg/mL of CRP. (B) Simplagrin (1 µM) shows no inhibitory effect in platelet aggregation induced by other agonists. PMA, phorbol 12-myristate 13-acetate (0.5 µM), ADP (10 µM), convulxin (100 pM), thrombin receptor activating peptide (TRAP) (5 µM), U46619 thromboxane A_2_ analog (0.7 µM), arachidonic acid (Ara acid) (1.5 mM), epinephrine (50 µM), ristocetin (1 mg/mL), and thrombin (0.1 U/mL).

Because collagen is recognized as the most thrombogenic component of the subendothelial matrix and the collagen-binding activity displayed by Simplagrin, we investigated whether Simplagrin has any effect in collagen-induced platelet aggregation. As shown in Figure 7A, Simplagrin (1 µM) inhibited human platelet-rich plasma (PRP) aggregation induced by a lower concentration of collagen (1 µg/mL), with 0.16 µM causing only a small delay in the shape change of platelets; however, Simplagrin (1 µM) failed to inhibit platelet aggregation when a higher dose of collagen (10 µg/mL) or collagen-related peptide (CRP) (5 µg/mL) was utilized as platelet aggregation agonist. Simplagrin (1 µM) shows no effect in platelet aggregation induced by other agonists, including the thromboxane A_2_ analog U46619 and CRP (Figure 7B). Although the current evidence suggests that the primary signaling of collagen induced platelet aggregation occurs via GPVI, other studies have shown that platelet response to low collagen concentrations is highly aspirin sensitive and therefore thromboxane A_2_ mediated [20]. Our results suggest that Simplagrin inhibition of collagen-induced platelet aggregation does not involve direct blocking of GPVI-collagen interaction. Rather, the inhibition might be due to steric hindrance, assuming that Simplagrin binds to a collagen region other than that to which GPVI binds.

### Simplagrin displays similar affinity for RGQPGVMGF peptide and soluble collagens

**Figure 8:**
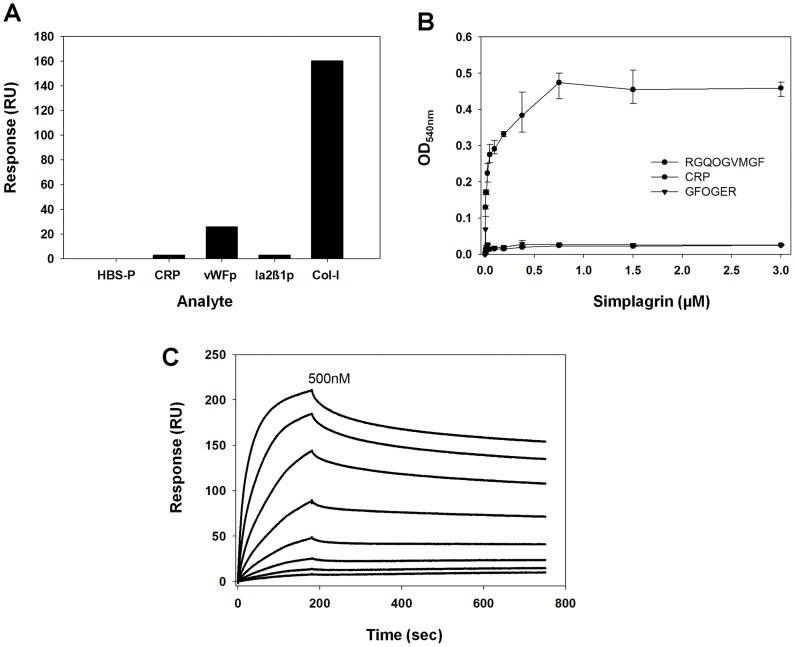
Simplagrin binds to RGQOGVMGF, the von Willebrand Factor (vWF) binding site on collagen. (A) Surface plasmon resonance binding analysis of immobilized Simplagrin with CRP (cross-linked GPO_10_), vWFpep (cross linked RGQOGVMGF), Iα_2_β_1_pep (cross-linked GFOGER) and Col-I (collagen type I). (B) Solid phase binding assay shows that Simplagrin binds in a dose response manner to wells coated with RGQOGVMGF peptides. Binding was detected using rabbit anti-Simplagrin antibodies. (C) Kinetic analysis showing that Simplagrin displays high affinity (K_D_ 11.1±0.59 nM) for RGQOGVMGF peptide. Analyte concentration ranging from 1–500 nM of RGQOGVMGF were flowed over immobilized Simplagrin at 30 µL/minute for 180 seconds, and the complex dissociation was monitored for 600 seconds before regeneration of the sensor surface. A global 1∶1 reaction model was used to calculate kinetic parameters.

Multiple collagen receptors have been identified on the platelet surface, namely GPVI, integrin α_2_β_1_, and GPIbα through collagen bound vWF complex [21]–[23]. In an attempt to identify the collagen region targeted by Simplagrin, SPR- and ELISA-based assays were designed. For SPR analysis, Simplagrin was immobilized on a CM5 sensor chip, and synthetic collagen-derived peptides were flowed over the sensor for 90 seconds at 20 µL/minute. As a positive control, collagen type I was also flowed over immobilized Simplagrin. Figure 8A shows that Simplagrin specifically binds to collagen (positive control) and the vWF collagen receptor peptide RGQOGVMGF; however, no detectable binding was observed when CRP [(GPO)_10_] or Iα_2_β_1_ (GFOGER) collagen-derived peptides were flowed over immobilized Simplagrin. For solid-phase analysis, a 96-well plate was coated with collagen type I (positive control) or RGQOGVMGF collagen-derived peptide. Wells were incubated with different concentrations of Simplagrin, and the binding capacity of Simplagrin to these two molecules was detected with purified rabbit anti-Simplagrin antibodies as described in Methods. Identical results were obtained using this method, confirming that Simplagrin specifically binds to RGQOGVMGF collagen-derived peptide (Figure 8B). Next we calculated the Simplagrin RGQPOVMGF affinity constants using SPR. To compare affinity of Simplagrin RGQOGVMGF peptide to that of Simplagrin collagen, kinetic analysis was carried out. For this experiment, the ka, kd, and K_D_ constants were calculated with Simplagrin as ligand and RGQOGVMGF peptide as analyte (Table 4). Figure 8C shows the sensograms of a typical kinetic experiment. The calculated K_D_ value (11.1±0.59 nM) for Simplagrin-RGQOGVMGF collagen-derived peptide interaction is in the same order of magnitude as that of Simplagrin collagens (Table 4). These results show that Simplagrin specifically binds to the RGQOGVMGF sequence in collagen.

**Table 4:** Surface plasmon resonance analysis of Simplagrin-RGQOGVMGF peptide.

	ka (1/Ms)	kd (1/s)	K_D_ (nM)
RGQOGVMGF peptide	7.269±0.97E+5	4.796±1.3E-4	11.104±0.59

Kinetic and binding constants were calculated using a 1:1 interaction binding model. Experiments were carried out in triplicate.

### “In solution” competition assay shows that Simplagrin blocks vWF-collagen interaction but not GPVI-collagen interaction

**Figure 9:**
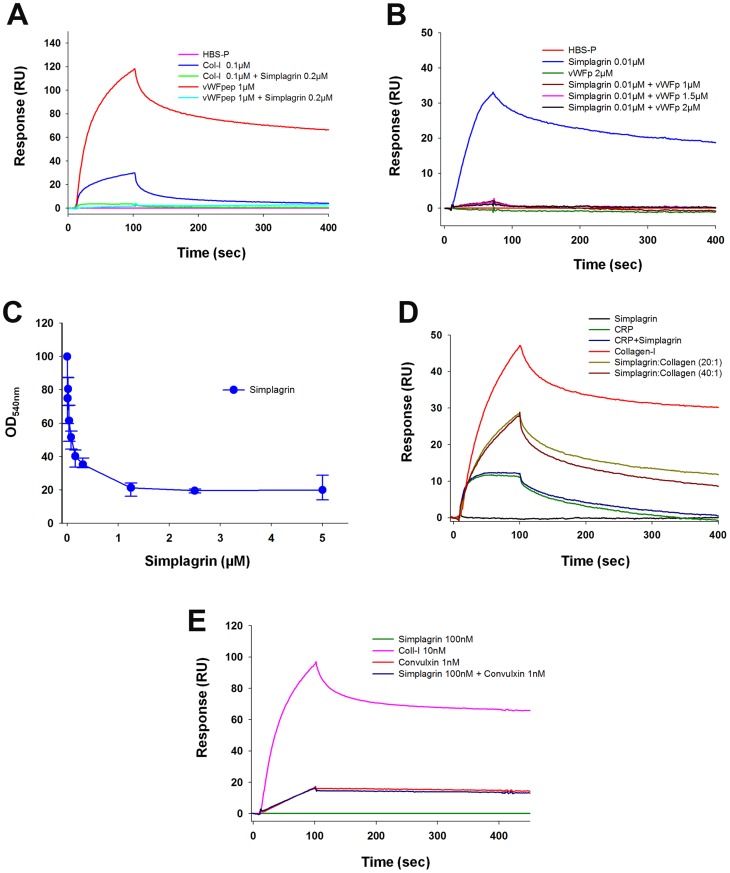
Simplagrin blocks von Willebrand Factor (vWF) interaction to collagen but not GPVI to collagen. (A) In solution competition using surface plasmon resonance (SPR) shows that Simplagrin inhibits interaction of collagen and RGQOGVMGF peptide on immobilized vWF. Collagen type I (0.1 µM) or RGQOGVMGF peptide (1 µM) in the presence or absence of Simplagrin (0.2 µM) were flowed over immobilized vWF. No detectable Simplagrin-vWF binding was observed. (B) Preincubation Simplagrin with saturating concentrations of RGQOGVMGF peptide abrogates Simplagrin-collagen interaction in SPR experiments. (C) Solid-phase assay showing that Simplagrin blocks, in a dose-response manner, collagen vWF interaction. (D) Simplagrin partially blocks GPVI-collagen interaction. SPR in solution competition shows that preincubation of collagen or CRP with Simplagrin at 1∶20 or 1∶40 molar ratios only reduces the response binding of collagen to GPVI approximately 60%; however, Simplagrin fails to block CRP-GPVI interaction. This can be explained by steric hindrance of Simplagrin binding to RGQOGVMGF sequence in collagen. (E) Control experiment showing that Simplagrin does not affect convulxin-GPVI interaction. All SPR experiments were carried out in triplicate.

Because collagen-derived peptide RGQOGVMGF and collagen were reported to be specific ligands for vWF, we determined the ability of Simplagrin to compete with collagen for vWF binding. For this assay, vWF was immobilized on a CM5 sensor chip, and collagen and RGQOGVMGF peptide were allow to interact with immobilized vWF in the presence or absence of saturating concentrations of Simplagrin. Figure 9A shows that Simplagrin can indeed block the interaction of collagen or RGQOGVMGF to immobilized vWF. No direct binding of Simplagrin to vWF was detected. Alternatively, Simplagrin was preincubated with saturating concentrations of RGQOGVMGF peptide and flowed over immobilized collagen type III. Figure 9B shows that Simplagrin binding to collagen is abrogated in the presence of RGQOGVMGF peptide indicating that Simplagrin binding site in collagen is the RGQOGVMGF sequence. As an orthogonal method, an ELISA was carried out to investigate the vWF collagen-blocking capacity of Simplagrin. For this experiment collagen-coated wells were preincubated with different concentrations of Simplagrin before vWF was added to each well. The blocking assay was assessed by the reduction of bound anti-vWF antibodies. Figure 9C shows that Simplagrin blocks, in a dose-response manner, the interaction of vWF collagen, with a calculated IC_50_ of approximately 0.1 µM. Finally, SPR was used to investigate whether Simplagrin interferes with collagen-GPVI interaction. For this experiment, GPVI was immobilized on a CM5 sensor chip followed by injection of collagen I or CRP, preincubated with or without saturating concentrations of Simplagrin. Figure 9D shows that Simplagrin does not completely abrogate collagen binding to immobilized GPVI even at 1∶20 and 1∶40 collagen:Simplagrin molar ratios. Furthermore, Simplagrin failed to block CRP-GPVI interaction. On the other hand, convulxin GPVI interaction was not affected by presence of Simplagrin (Figure 9E). No direct binding of Simplagrin to GPVI was detected. This result further supports that Simplagrin binds preferentially to the RGQOGVMGF sequence in collagen and also explains the lack of platelet aggregation inhibition at higher concentrations of fibrillar collagen (10 µg/mL). This competition assay reinforces the hypothesis of steric hindrance of platelet aggregation inhibition of Simplagrin at low concentrations of fibrillar collagen.

### Inhibition of platelet adhesion to collagen under high-shear stress

**Figure 10:**
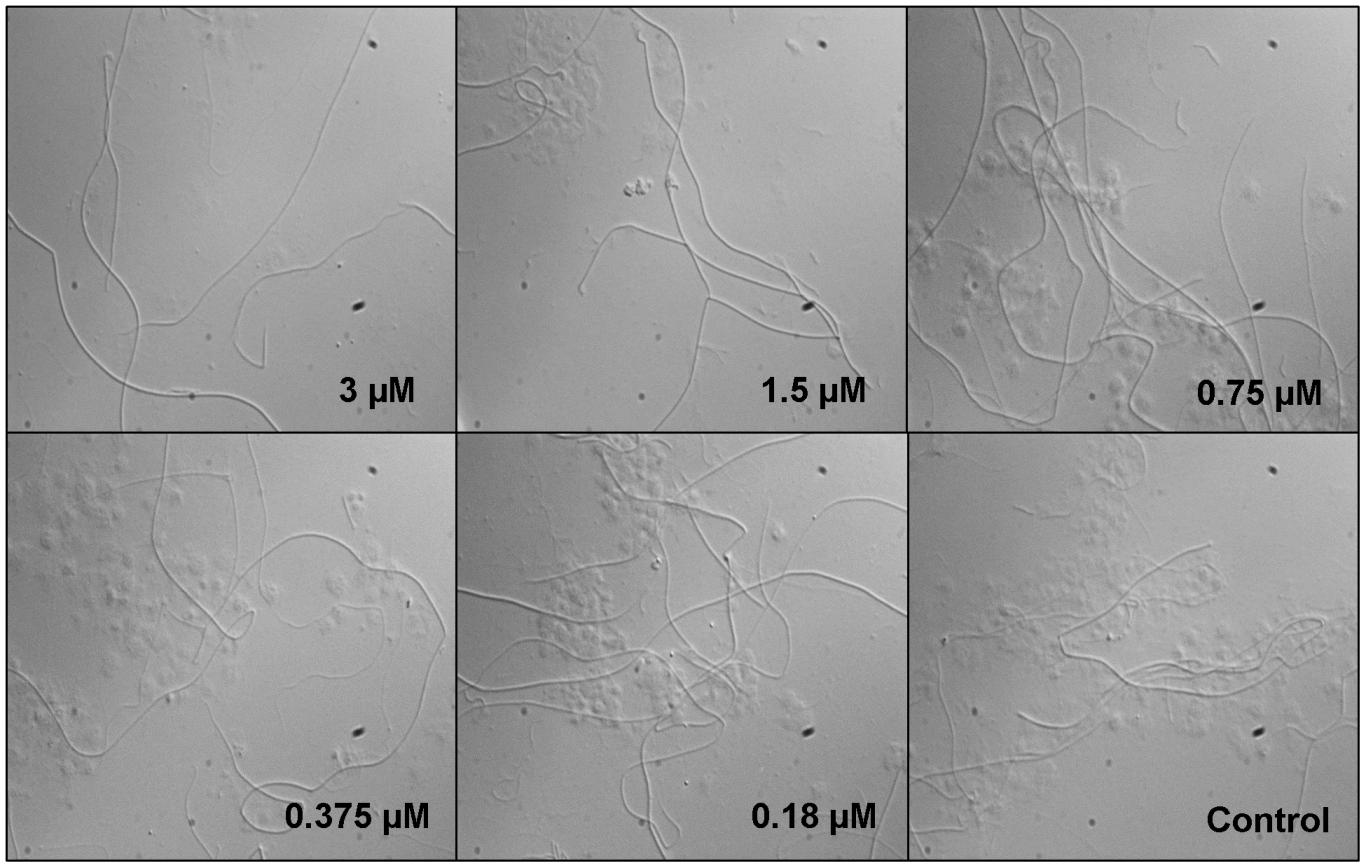
Effect of Simplagrin on platelet adhesion to fibrillar collagen under high shear stress. Simplagrin inhibits platelet collagen interaction under high shear stress in a dose dependent fashion. Anticoagulated whole blood from healthy patients was perfused over immobilized fibrillar collagen for 240 seconds at a shear rate of 1500^−1^ in the presence of different doses of Simplagrin and immediately perfused with Tyrode's buffer at the same shear rate to remove loosely bound platelets. Coverslips were mounted and analyzed under bright-field microscopy. Representative results of a typical experiment (n = 6).

The initial steps in the hemostatic cascade include platelet interaction with the exposed extracellular matrix at sites of injury as well as ADP released by damaged cells. On vascular injury at sites of high-shear rates, the platelet integrin GPIbα interacts with collagen-bound vWF to initiate the tethering of circulating platelets to the vessel wall. Tethered platelets subsequently roll on the damaged vessel wall, a process that is amplified by activation of platelet integrin GPIIb/IIIa [24]. Because Simplagrin blocks collagen vWF interaction, we investigated whether Simplagrin was able to reduce platelet adhesion to collagen under high wall shear stress. For this experiment, anticoagulated whole blood containing different concentrations of Simplagrin (0, 0.18, 0.37, 0.75, 1.5 and 3 µM) was perfused for 180 seconds over fibrillar collagen coated coverslips at high shear rates (1500 s^−1^) followed by continuous perfusion of Tyrode's buffer for 180 seconds at the same shear rate. Figure 10 shows that adhesion of platelet to fibrillar collagen was reduced in a dose-dependent manner with an IC_50_ of approximately 0.75 µM, with complete abrogation of platelet tethering to collagen fibers at 3 µM. This result is in agreement with the SPR and solid-phase analyses showing that Simplagrin inhibits collagen vWF interaction under static conditions. We did not find any effect on platelet adhesion under static conditions using calcein-labeled platelets (not shown).

### Simplagrin reduces occlusive thrombus formation time *in vivo* without compromising hemostasis

**Figure 11:**
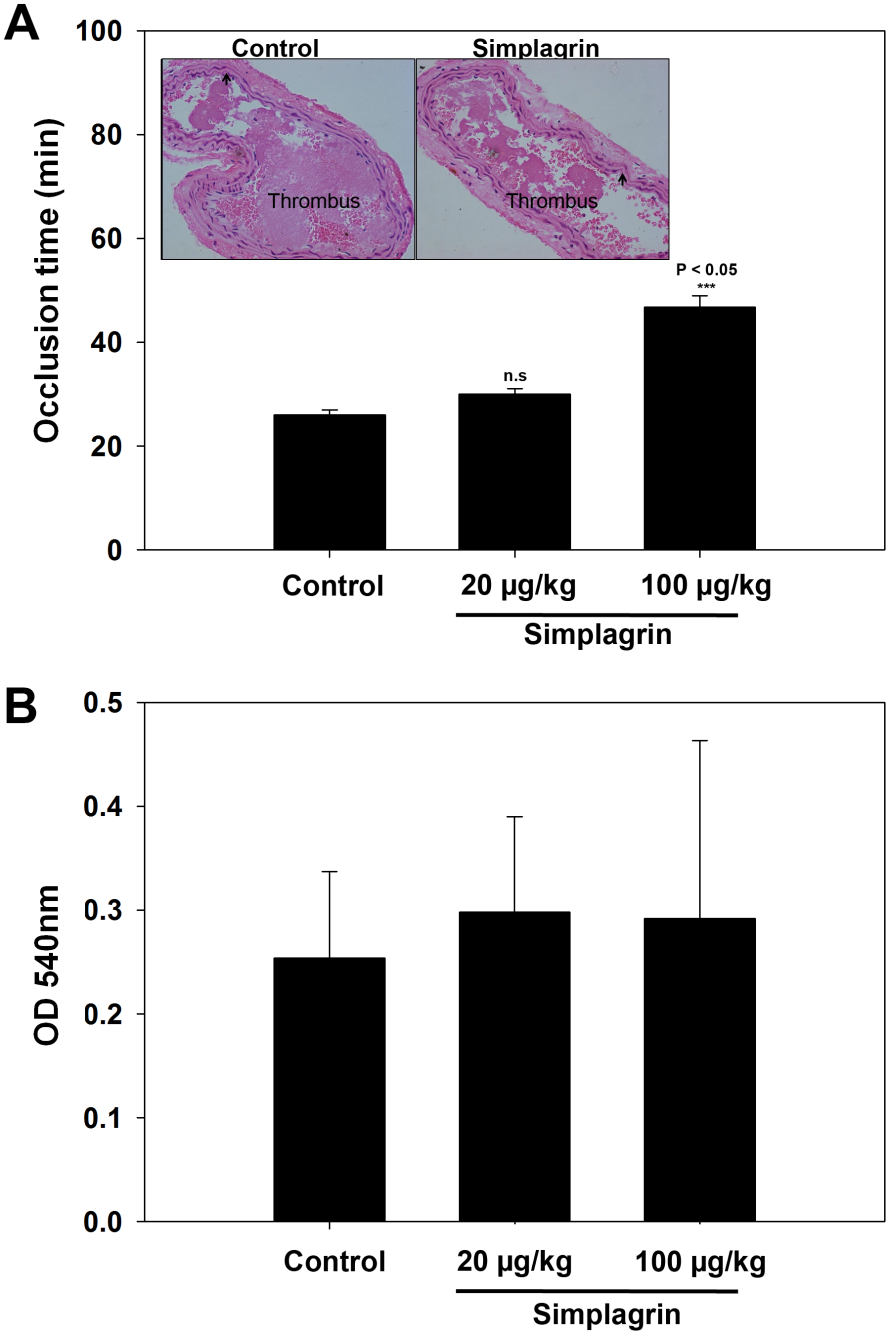
Effects of Simplagrin on thrombosis and bleeding. (A) Simplagrin prevents thrombus formation *in vivo*. Photochemical induced carotid artery injury: female mice were treated with PBS (control) and two different doses (20 and 100 µg/kg) of Simplagrin 15 minutes before thrombosis was induced, and blood flow was measured as described in Methods. Inset: Hematoxylin and eosin stained cross-sections of injured carotid artery from mice treated with PBS and Simplagrin (100 µg/kg). Arrows show the collagen in the arteries. (B) Simplagrin does not increase bleeding in tail transection assay. Mice (n = 5) were intravenously treated with PBS (control) or Simplagrin (20 and 100 µg/kg). After 15 minutes, the distal 2 mm of the tail was surgically removed and blood loss monitored for 30 minutes. Mean and SEM are shown. P<0.05 was considered statistically significant.

The antithrombotic effect of Simplagrin was investigated using a photochemically induced thrombosis model in C57BL*/*6 mice. Figure 11A shows that arterial blood flow of control mice treated with PBS alone stopped in 26±1.78 minutes (25–29 minutes). On the other hand, mice treated with Simplagrin at 20 and 100 µg/kg had their blood flow stopped at 30±2.09 (26–30 minutes) and 46.8±4.4 min (42–55 minutes), respectively. The prolongation of time to arterial occlusion was statistically significant (P<0.05) at 100 µg/kg. Considering that the most widely used anti-platelet agents (e.g., aspirin, heparin, and Clopidogrel, among others) carry the risk of increased bleeding rate, we investigated whether Simplagrin can increase the bleeding rate in mice. Evaluating the effect of Simplagrin in tail-bleeding assays would provide an additional measure of its antihemostatic effect *in vivo*. Tail bleeding was determined in BALB/c mice after intravenous injection of PBS (control) or Simplagrin (20 and 100 µg/kg). No significant difference in tail bleeding was found in mice treated with Simplagrin or PBS (Figure 11B). Our results demonstrate that Simplagrin can inhibit collagen-induced platelet aggregation and adhesion without affecting general hemostasis. Although platelet aggregation inhibition in general can cause prolongation of bleeding time, mouse tail bleeding is highly sensitive to levels of coagulation factors [25].

## Discussion

For blood feeding arthropods, the vertebrate hemostatic responses represent an important barrier for acquiring a successful blood meal. For this reason, blood feeders have evolved salivary secretions rich in molecules that affect hemostasis, including vasodilators and inhibitors of blood clotting and platelet aggregation. Among the platelet inhibitors, antagonists of collagen induced platelet aggregation and adhesion have been found in SG of ticks and other hematophagous animals (reviewed in [2]).

It was recently reported that *An. stephensi* and *Ae. aegypti* each express a salivary collagen-binding protein that inhibits collagen-induced platelet aggregation and adhesion. Both molecules (AAPP from *An. stephensi* and Aegyptin from *Ae. aegypti*) were shown to block collagen interaction with its three major ligands, GPVI, vWF, and integrin α_2_β_1_ [10]–[12]. The Aegyptin family of salivary proteins has also been described in the Diptera suborder Nematocera, where anophelines and *Simulium* appear to have a single gene coding for this family, while culicines have multiple genes [2]. Comparisons between *Anopheles*, *Culex*, *Aedes*, and now *Simulium* sialomes (from the Greek sialo  =  saliva) showed that each species contained genus-specific salivary protein families and even subgenus-specific families, suggesting that the evolution of salivary proteins has occurred at a very fast pace, possibly caused by the immune pressure of their hosts [26].

The mechanism of action of Simplagrin can be explained by its binding to collagen, blocking vWF-collagen interaction. By blocking this interaction, Simplagrin ensures a delay in platelet activation and aggregation, especially in small arteries and arterioles where shear force is rather high (e.g., 1500s^−1^) and the interaction between GPIb complex and vWF is crucial.

A possible explanation for the platelet aggregation-inhibition effect of Simplagrin at low concentrations of fibrillar collagen can be proposed on the basis that collagen has multiple binding sites for GPVI, vWF, and integrin α_2_β_1_, all of them mapped in close proximity [23]. The complexity of fibrillar collagen organization may also facilitate interaction between receptors bound to adjacent or nearby collagen monomers within a fiber [23]. Taking into consideration that Simplagrin displays an elongated, non-globular conformation, steric hindrance seems to be responsible for its platelet aggregation inhibition at low collagen concentrations. Interestingly, the C-terminal domain of Simplagrin also binds to collagen but with significantly lower affinity for collagen type I and III (K_D_ 284.7±23.8 nM and 162.3±5.16 nM, respectively) when compared to that of Simplagrin to collagen I and III (K_D_ 5.6±0.52 nM and 2.1±0.35 nM, respectively). As expected from this low affinity for collagens, the C-terminal domain was unable to inhibit collagen-induced platelet aggregation; however, it causes a delay in the shape change in platelets activated with collagen (Figure S1). These results are in agreement previously published work demonstrating that the C-terminal domain of Aegyptin and AAPP is responsible for collagen binding and platelet aggregation inhibition[11], [27].

The overall difference in platelet aggregation and adhesion inhibition between Simplagrin and Aegyptin (Table 5) could be explained by the conformation change occurring upon collagen-Aegyptin interaction (Figure 7A and B). “In solution” analysis results showed that collagen undergoes unwinding caused by Aegyptin as determined by CDS; however, no significant conformational change was detected in Simplagrin-collagen complex. Fibrillar collagen can assemble stable triple helices, which in turn can form a complex 3D fibrous superstructure needed for platelet receptor binding to collagen and platelet activation (reviewed in [22],[28]). It was proposed that blood feeding success through inhibition of platelet aggregation is a vital salivary function in blood feeding arthropods [29]. These authors hypothesized that faster probing and feeding times would reduce the duration of vector-host contact and hence increase survival of the feeder. In this scenario, unwinding of collagen by Aegyptin may represent an evolutionary advantage over Simplagrin, making it a more efficient inhibitor and resulting in shorter probing and feeding time in *Ae. aegypti* mosquitoes [30]; however, the resulting antihemostatic effect of saliva—deriving from dozens of protein families, many still with unknown function—should not be attributed to a single molecule.

**Table 5:** Summarized comparison between Aegyptin and Simplagrin main features.

Feature	Aegyptin	Simplagrin
Non-globular, elongated conformation	Yes	Yes
Collagen-binding	Yes	Yes
Collagen-induced platelet aggregation inhibition	Yes	Yes
vWF-collagen blocking	Yes	Yes
Inhibition of thrombus formation *in vivo*	Yes	Yes
Increase of tail bleeding	No	No
Inhibition of platelet adhesion to collagen	Static/Flow	Flow
Blocking GPVI- and Iα_2_β_1_-collagen interaction	Yes	No
Involves conformational change of collagen	Yes	No

It has also been proposed that Culicidae and Simuliidae families diverged in the middle Triassic, sharing a common blood- (or insect hemolymph)- feeding ancestor approximately 250 MYA. Accordingly, this common ancestor originated before the irradiation and expansion of birds in the Jurassic (200 MYA) and of mammals 60 MYA. As the Culicomorpha evolved to produce mosquitoes (Culicidae), frog-feeding flies (Corethrellidae), biting midges (Ceratopogonidae), and black flies (Simuliidae), land vertebrates evolved to produce birds and mammals. Within this scenario, the blood feeding sialome evolved within each fly species in concert with their vertebrate “hemostome” and “immunome,” the latter battering the salivary proteins by either neutralizing their function or creating inflammatory reactions such as pain or itching that, in concert with host behavioral defenses, could disrupt feeding. This scenario of concerted “birth and death” evolution of multigene families [31] could explain the evolutionary speed of the blood feeding sialome as well as the recruitment or exaptation of new gene families [15]. Perhaps a collagen-binding salivary protein was an evolutionary innovation present in an ancient dipteran ancestor that evolved into the current Aegyptin-like protein family commonly found in sialomes of Culicomorpha, excluding *Corethrella appendiculata* [32]. It might be possible that this salivary function has been substituted by a different gene family in *C. appendiculata* SGs.

In conclusion, our results show that Simplagrin is a nonglobular, elongated collagen-binding protein that prevents platelet adhesion under high shear stress. Simplagrin binds directly to the von Willebrand binding sequence RGQOGVMGF in collagen, retaining the same biologic function as the mosquito Aegyptin-like proteins but with a distinct mechanism of inhibition. Despite the low similarity between Simplagrin and Aegyptin at the amino acid level, the possibility of convergent evolution of the Aegyptin family cannot be excluded since the proposed the mechanism(s) of action differ between both molecules. Due the overall similarity in the modelled 3D structure of Simplagrin and Aegyptin (Figure S2), it might be argued that even structures could have converged at this low level of amino acid identity. Our results support the common origin of hematophagy in mosquitoes and black flies as proposed by Grimaldi and Engel [16], with the presence of a unique protein family with conserved primordial function found in black flies and mosquitoes.

## Supporting Information

Figure S1 **Simplagrin carboxy-terminus domain (Simplagrin-CT) is responsible for its collagen binding activity.** (A) Purification of Simplagrin-CT was carried out by Hitrap chelating column followed by size exclusion chromatography. (B,C) Simplagrin-CT displays a significantly lower binding affinities for Collagen type I and III as measured by surface plasmon resonance. (D) Effect of Simplagrin-CT on collagen-induced platelet aggregation. Platelet aggregation was estimated by turbidimetry under stirring conditions at 37°C. Simplagrin-CT only causes a delay in the shape-change phase of collagen induced platelet aggregation.
(TIF)

Figure S2 **Simplagrin-CT and Aegyptin C-terminal domains are structurally similar.** (A) Three-dimensional structure prediction of Simplagrin-CT and Aegyptin C-terminal domain showing a ribbon diagram of the model generated using PyMOL. Coordinates were generated automatically by I-TASSER software. (B) Surface density map of Simplagrin-CT and Aegyptin C-terminal domain generated by PyMOL APBS tools. Electrostatic potential surfaces of the model showing the positively (blue) and negatively (red) charged surfaces.
(TIF)

Text S1 Supporting Methods.
(DOCX)
