# Supplementary figures and images for: Simplagrin, a Platelet Aggregation Inhibitor from Simulium nigrimanum Salivary Glands Specifically Binds to the Von Willebrand Factor Receptor in Collagen and Inhibits Carotid Thrombus Formation In Vivo
Source: PLoS Negl Trop Dis. 2014 Jun 12;8(6):e2947. doi: 10.1371/journal.pntd.0002947 (PMC4055580; doi:10.1371/journal.pntd.0002947)

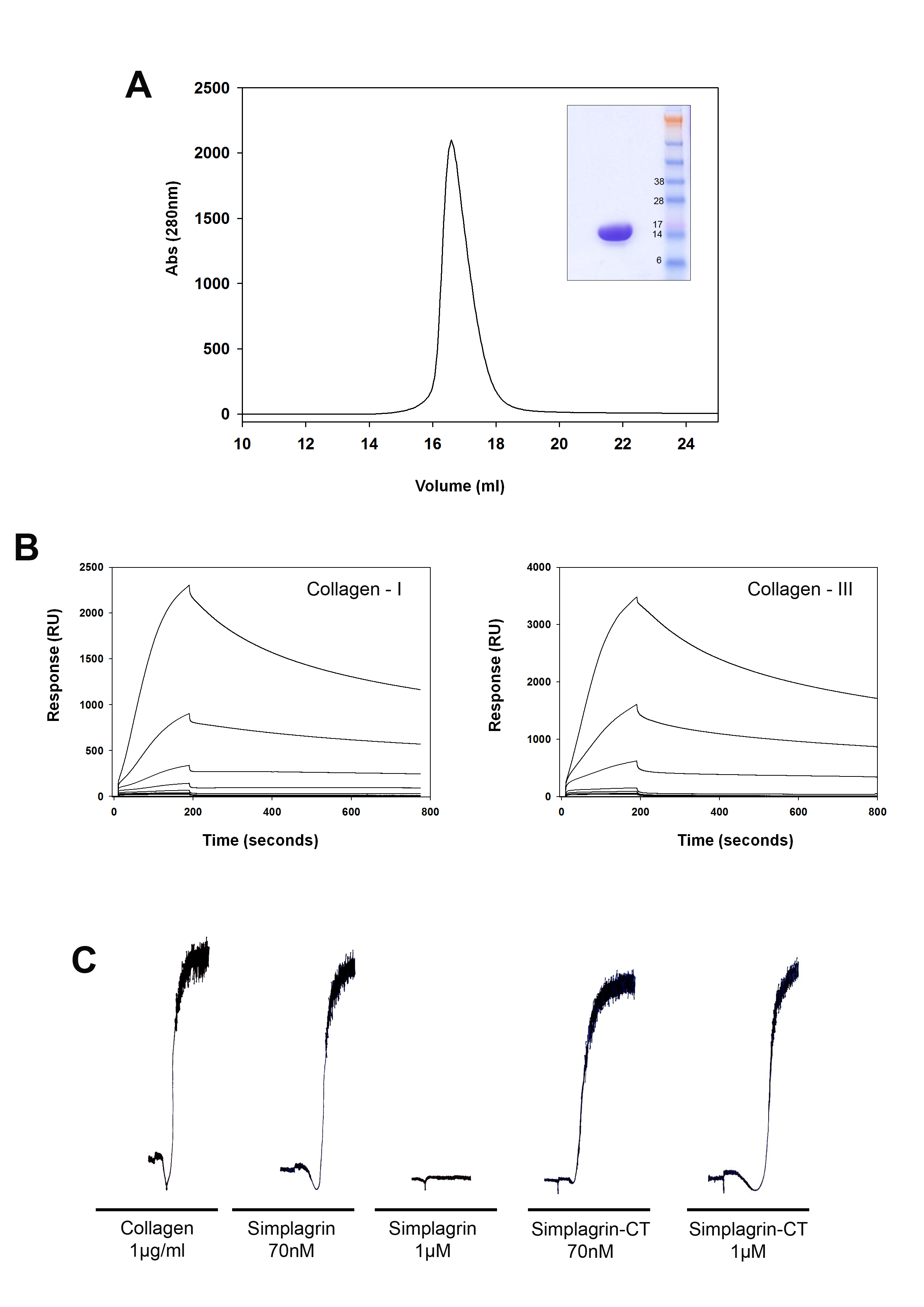

Supplement: Figure S1 — Simplagrin carboxy-terminus domain (Simplagrin-CT) is responsible for its collagen binding activity. (A) Purification of Simplagrin-CT was carried out by Hitrap chelating column followed by size exclusion chromatography. (B,C) Simplagrin-CT displays a significantly lower binding affinities for Collagen type I and III as measured by surface plasmon resonance. (D) Effect of Simplagrin-CT on collagen-induced platelet aggregation. Platelet aggregation was estimated by turbidimetry under stirring conditions at 37°C. Simplagrin-CT only causes a delay in the shape-change phase of collagen induced platelet aggregation. (TIF) [file pntd.0002947.s001.tif]

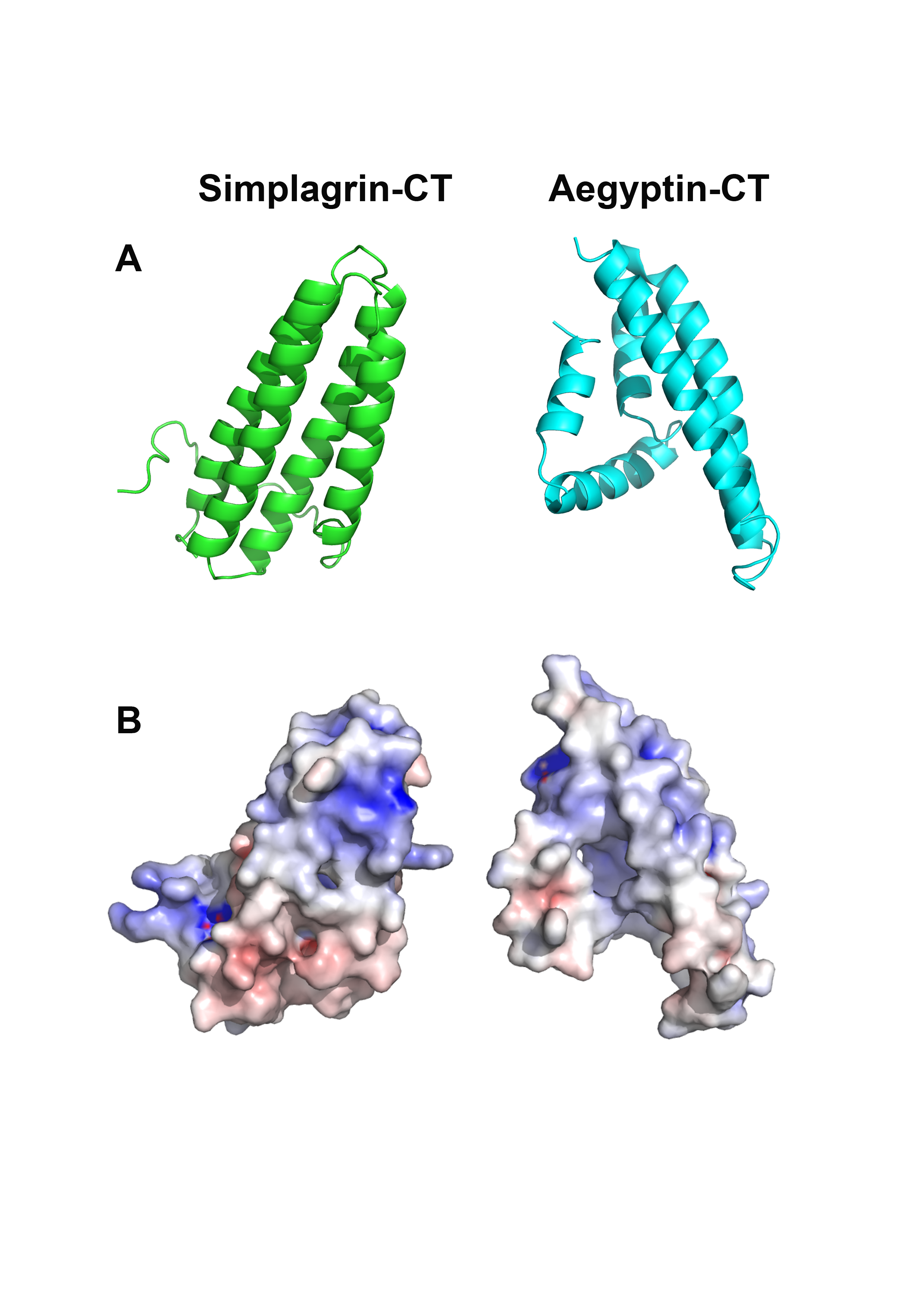

Supplement: Figure S2 — Simplagrin-CT and Aegyptin C-terminal domains are structurally similar. (A) Three-dimensional structure prediction of Simplagrin-CT and Aegyptin C-terminal domain showing a ribbon diagram of the model generated using PyMOL. Coordinates were generated automatically by I-TASSER software. (B) Surface density map of Simplagrin-CT and Aegyptin C-terminal domain generated by PyMOL APBS tools. Electrostatic potential surfaces of the model showing the positively (blue) and negatively (red) charged surfaces. (TIF) [file pntd.0002947.s002.tif]
