# Supplementary material for: Simplagrin, a Platelet Aggregation Inhibitor from Simulium nigrimanum Salivary Glands Specifically Binds to the Von Willebrand Factor Receptor in Collagen and Inhibits Carotid Thrombus Formation In Vivo
Source: PLoS Negl Trop Dis. 2014 Jun 12;8(6):e2947. doi: 10.1371/journal.pntd.0002947 (PMC4055580; doi:10.1371/journal.pntd.0002947)
Supplement: Text S1 — Supporting Methods. (DOCX) [file pntd.0002947.s003.docx]

*Cloning, expression and purification of Simplagrin C-terminal domain*. PCR fragment coding for the C-terminal domain of Simplagrin (Simplagrin-CT) was amplified (Platinum HiFi Supermix, Invitrogen) from a plasmid construct containing the Simplagrin ORF (VR2001-Sim50). Domain-specific primers for the C-terminal domain were: CterSimplagrinF 5’- CATATGAAGAGTAAAGGTAAAGGCGCCAAGG-3’; and CterSimplagrinR v 5’- CTCGAGTTAGTGGTGGTGGTGGTGGTGATAGTAGCCCCCTTGAAGGCAGG-3’. The reversed primer was designed to have a 6xHis-tag followed by a stop codon. The amplified PCR fragment was digested with NdeI and XhoI restriction enzymes (Life Technologies) and directionally cloned into pET17b. Recombinant protein expression, refolding and purification were carried out as described before [[1](#_ENREF_1)]. N-terminal sequence of Simplagrin-CT was verified by Edman degradation. Collagen-binding assay and platelet aggregometry were carried out as describe in Methods section.

**Reference**

1. Calvo E, Mans BJ, Andersen JF, Ribeiro JM (2006) Function and evolution of a mosquito salivary protein family. J Biol Chem 281: 1935-1942.
